# An Update on the Pivotal Roles of Probiotics, Their Components, and Metabolites in Preventing Colon Cancer

**DOI:** 10.3390/foods12193706

**Published:** 2023-10-09

**Authors:** Xue Deng, Jing Yang, Yu Zhang, Xiaoyong Chen, Chen Wang, Huayi Suo, Jiajia Song

**Affiliations:** 1 College of Food Science, Southwest University, Chongqing 400715, China; dx15708131969@email.swu.edu.cn (X.D.); y000063@swu.edu.cn (Y.Z.); cxy0520@swu.edu.cn (X.C.); wangchen20220307@swu.edu.cn (C.W.); birget@swu.edu.cn (H.S.); 2 Chongqing Engineering Research Center for Processing & Storage of Distinct Agricultural Products, Chongqing Technology and Business University, Chongqing 400067, China; jyang@ctbu.edu.cn; 3 National Citrus Engineering Research Center, Southwest University, Chongqing 400712, China

**Keywords:** probiotics, colon cancer, colon cancer treatment, anti-cancer mechanisms

## Abstract

Diet, lifestyle, and gut microbiota composition are key risk factors for the progression of colon cancer. Probiotics are living microorganisms that can offer health benefits to the parasitifer when ingested in competent quantities. Several in vivo, in vitro, and clinical studies have demonstrated that probiotics can prevent and mitigate the development of colon cancer. The anti-colon cancer mechanisms of probiotics include the suppression of cell proliferation and the promotion of cancer cell apoptosis, immunomodulation, the modulation of intestinal microorganisms and their metabolism, strengthening the intestinal barrier, and antioxidant effects. This article describes the pathogenesis of colon cancer and the available therapeutic options. In addition, this paper reviews the mechanisms by which probiotics mitigate colon cancer as well as the mitigating effects of probiotic components and metabolites on colon cancer.

## 1. Introduction

Colon cancer is the third most common cancer worldwide. Being a malignant tumor, the incidence of and mortality rate associated with colon cancer are currently high worldwide [1]. According to statistics, the incidence of colon cancer in 2020 was 10% and the associated mortality rate was 9.4% [2]. Most colon cancers could be attributed to lifestyle and aging, whereas a few cases are due to underlying genetic disorders. Other factors, such as dietary habits and gut microbiota, are also related to colon cancer development [3,4].

Conventional therapeutic strategies for colon cancer comprise surgical removal, chemotherapy, and radiotherapy. Surgery is usually indicated in patients whose colonic lesions are early and localized. Recovery is expected after surgical removal, which is one of the efficacious treatments for localized cancer [5]. On the contrary, those with metastatic colon cancer tend to opt for chemotherapy as their primary treatment. Radiotherapy, which is yet another treatment modality, uses ionizing radiation to inhibit the multiplication of malignant cells and can effectively address prevalent forms of cancer, including skin cancers, lung carcinomas, lymphomas, head and neck carcinomas, among others [5,6]. Immunotherapy is a new therapeutic strategy for colon cancer, and its efficacy has been proven in clinical trials for patients with colon cancer, especially those with high microsatellite instability (MSI-H) [7]. However, some of the current treatments exert several adverse effects on the body; hence, exploring new alternatives for the prevention and treatment of colon cancer becomes necessary.

The World Food and Agriculture Organization has provided a definition of probiotics as “living microorganisms that, when administered in adequate quantities, provide health benefits to the host”. There is growing evidence to support the potential role of probiotics in colon cancer prevention. In vitro experiments have demonstrated the proliferation suppression of colon cancer cells by various probiotics. *Pediococcus pentosaceus* GS4, *Pediococcus pentosaceus* SP2, and *Lacticaseibacillus paracasei* SP5 are a few examples [8,9]. Administration of *Lacticaseibacillus rhamnosus* GG and *Lactobacillus acidophilus* to mice with colon cancer has been shown to reduce tumor incidence [10]. *Lacticaseibacillus rhamnosus* and *Lactobacillus acidophilus* have been reported to alleviate bowel symptoms and enhance the quality of life of patients with colon cancer [11]. In addition, probiotic metabolites and bacterial fractions can also offer resistance to colon cancer. Cell-free supernatant of *Lacticaseibacillus rhamnosus* has been documented to inhibit the metastasis and proliferation of HT-29 cells and promote their apoptosis [12]. Furthermore, probiotic extracellular polysaccharides (EPSs) and S-layer protein (Slp) can inhibit colon cancer [13,14].

This review summarizes the pathogenesis and treatment of colon cancer and also examines the recent advances in probiotics as well as the mechanisms by which they alleviate colon cancer. In addition, the progress in studies pertaining to the inhibition of colon cancer by probiotic components and related metabolites is presented.

## 2. Risk Factors of Colon Cancer

### 2.1. Diet

Epidemiologic and experimental findings often link food and nutrient intake to colon cancer risk. Calcium occurs in all types of foods, and increasing its intake may reduce colon cancer risk [15]. Moreover, according to epidemiological studies, vitamin D deficiency is yet another factor associated with an increased incidence of colon cancer that also adversely affects the survival of patients with colon cancer [16]. A meta-analysis revealed that vitamin D intake has been clinically associated with improved long-term survival in patients with colon cancer [17]. Moreover, dietary fiber and whole grain intake exerts a positive effect on cancer prevention [18]. On the contrary, excessive intake of red and processed meats significantly increases the risk of colon cancer [19]. Heme iron in red meat leads to the formation of endogenous N-nitroso compounds as well as heterocyclic amino acids and polycyclic aromatic hydrocarbons when cooked at high temperatures. The high intake of all these foods has been linked to colon tumors [20,21]. Sweets typically contain higher amounts of carbohydrates and fats. Furthermore, research indicates a significant correlation between a high consumption of sweets and an elevated risk of colon cancer [22]. Notably, Western dietary patterns characterized by increased intake of processed and red meat, refined grains, and sweets have been associated with the development of colon cancer [23,24,25,26]. Conversely, adopting a diet abundant in fruits, vegetables, dairy products, and whole-grain products has been linked to a reduced likelihood of colon cancer development [26,27].

### 2.2. Obesity

Obesity is characterized by chronic inflammation and leads to an increased risk of colon cancer as well as increased mortality and complications after colon cancer surgery [28]. Elevated levels of inflammatory factors, such as tumor necrosis factor-α (TNF-α), interleukin-1 (IL-1), and IL-6, have the potential to initiate colon carcinogenesis [29,30]. Obesity can trigger an increase in the levels of insulin and insulin-like growth factor 1 (IGF-1) in the blood [31]. Insulin and IGF-1 have significant implications in colon cancer development, as they activate the phosphatidylinositol 3-kinase (PI3K)/Akt signaling pathway, stimulating cell growth and cell cycle signaling [31]. Moreover, studies indicated that insulin and IGF-1 can enhance the proliferation of colon cancer cells and facilitate the growth of colon cancer allografts in obese mice [32,33]. Obesity has been correlated with higher levels of leptin, a hormone originating from adipose tissue. Leptin plays a crucial role in regulating the homeostatic balance of adipose tissue mass. Some studies have demonstrated that leptin can stimulate the growth of colon cancer cells [34,35]. Conversely, adiponectin, which enhances energy expenditure, can mitigate colon cancer related to obesity by inhibiting the mammalian target of the rapamycin (mTOR) pathway [36].

### 2.3. Gut Microorganisms

Colon cancer progression is intricately linked to ecological disturbances in the gut microbiota. Colon cancer is induced by oncogenic bacteria, such as enterotoxigenic *Bacteroides fragilis* (ETBF), via direct interactions with colonic epithelial cells and by altering the microbiota composition at the colonic site [37]. The toxin fragilysin produced by ETBF binds to colonic epithelial cells and in turn activates the proto-oncogene MYC and promotes the expression of the Wnt/β-linker proteins in the nucleus. These alterations induce the hyperproliferation of colonic epithelial cells [38,39]. ETBF may induce changes in the bacterial communities in the local environment of tumors and may permit the establishment of transient bacteria, such as *Fusobacterium nucleatum* [40]. *Fusobacterium nucleatum* stimulates the growth of colon tumor cells by triggering autophagy through the Toll-like receptor-4 pathway [41]. Escherichia coli inhabits the human gut and specific pathogenic strains have been linked to colon cancer. The production of colicin by *Escherichia coli* can lead to DNA damage and cell mutations, a phenomenon closely associated with the development of colon cancer [42,43]. Additionally, *Enterococcus faecalis* is capable of generating reactive oxygen species (ROS) and superoxide anions, which can induce DNA damage, potentially contributing to the development of cancer [38]. An increased abundance of *Bacteroides fragilis*, *Escherichia coli*, *Enterococcus faecalis*, *Fusobacterium nucleatum*, and *Streptococcus gallolyticus* and a decreased abundance of *Bifidobacterium*, *Clostridium*, *Faecalibacterium*, and *Roseburia* have been observed in patients with colon cancer [44]. A substantial reduction in butyrate-producing bacteria, such as *Lachnospiraceae*, among individuals with colon cancer, may result in an upsurge in pathogenic microorganisms. This, in turn, contributes to the progression of colon cancer [45]. Sulfidogenic bacteria, such as *Fusobacterium*, *Desulfovibrio*, and *Bilophila wadsworthia*, have been shown to induce genomic instability, which has been linked to colon cancer via the generation of hydrogen sulfide [46]. The intestinal microbiota participates in the metabolic transformation of the tumor and tumor-suppressor metabolites, which influences the development of colon cancer [47]. Secondary bile acids serve as predisposing agents for colon cancer. Gut microbes participate in the synthesis and transformation of these secondary bile acids, including lithocholic acid and deoxycholic acid [48]. Additionally, carnitine found in red meat produces trimethylamine, which, in collaboration with gut microbes, generates trimethylamine-n-oxide, a metabolite associated with the development of colon cancer [49].

### 2.4. Genetic Instability

The pathogenesis of colon cancer can be attributed to genetic or epigenetic changes, with genomic instability serving as the underlying process. In the pathogenesis of colon cancer, at least three pathways of genomic instability exist: chromosomal instability, microsatellite instability, and aberrant DNA methylation [50]. Colon cancer progresses through several stages that involve the gradual accumulation of mutations in many tumor suppressor genes and proto-oncogenes. Along with the activation of oncogenes, some tumor suppressor genes such as adenomatous polyposis coli (APC) and p53 are mutated and inactivated [51,52]. APC is recognized as a tumor suppressor protein that opposes the Wnt signaling pathway [53]. The inhibition of the Wnt signaling pathway has been linked to the effectiveness of certain dietary agents in preventing colon cancer [54]. Additionally, mutations in APC are closely associated with the initiation of colon tumorigenesis [55].

## 3. Treatment Options for Colon Cancer

### 3.1. Surgical Treatment

To date, the standard therapeutic option for colon cancer, which is a malignant tumor, is surgical removal. Surgical treatment can be classified as radical intervention and palliative. The former includes right or left hemicolectomy, segmental colectomy on transverse or sigmoid colon, and subtotal or total colectomy. The latter is categorized as colostomy and colonic bypass and is applied in advanced nonresectable stages [56].

### 3.2. Adjuvant Chemotherapy

Fluorouracil operates by inhibiting thymidylate synthase, an essential enzyme in DNA synthesis. Leucovorin, on the other hand, acts as a reductive folate, augmenting the inhibitory impact of fluorouracil on thymidylate synthase. For patients with colon cancer, fluorouracil combined with leucovorin (FL) was the earliest standard adjuvant therapy, and it reduced the likelihood of tumor recurrence [57]. Oxaliplatin, a third-generation platinum derivative, combats cancer by antagonizing DNA replication and transcription. Patients were treated with FL alone or in combination with oxaliplatin after radical resection for stage II or III colon cancer. The rate of disease-free survival at 3 years was 78.2% in the oxaliplatin-added group and 72.9% in the no-addition group. The occurrence rate of febrile neutropenia was 1.8% and that of adverse reactions in the gastrointestinal tract was low in the group to which oxaliplatin was added [58]. The use of oxaliplatin in combination with fluoropyrimidine significantly improved disease-free and overall survival in patients with stage III colon cancer with MSI [59]. Capecitabine, an oral prodrug of 5-fluorouracil (5-FU), undergoes conversion into 5-FU within the body, where it effectively hinders DNA synthesis. Postoperative therapy with capecitabine substantially decreased the likelihood of death and recurrence compared with patients treated using surgery only. Furthermore, a 16-week capecitabine chemotherapeutic regimen improved the survival in elderly patients with stage III colon cancer [60]. Patients receiving capecitabine and oxaliplatin had elevated disease-free survival rates and overall survival rates of 62% and 80%, respectively, at 2 years. In addition, the efficacy of the combination of capecitabine and oxaliplatin was comparable to that of 5-FU [61]. This finding implies that adjuvant therapy with capecitabine in combination with oxaliplatin is feasible in patients with early stage colon cancer. Irinotecan functions as an inhibitor of topoisomerase I, preventing DNA untangling and, in turn, disrupting DNA replication and cell division. In a meta-analysis of randomized controlled studies, the supplementation with irinotecan considerably enhanced the progression-free survival rate and median progression-free survival and reduced disease progression compared with FL. However, an increased incidence of grade ≥3 adverse events was observed with irinotecan treatment. These adverse reactions, which mainly involved diarrhea and neutropenia, were generally acceptable [62]. Bevacizumab is a targeted drug that obstructs the vascular endothelial growth factor, thereby decelerating tumor growth and enhancing the efficacy of chemotherapy. When bevacizumab was added to first-line chemotherapy in patients with metastatic colon cancer, progression-free survival was significantly improved. Bevacizumab monotherapy may be considered in certain circumstances, such as cumulative toxicity or patient refusal, especially in RAS wild-type patients [63].

### 3.3. Immunotherapy

In a study, patients with stage II and III colon cancer were treated with active specific immunotherapy (ASI) using autologous tumor cells and an immunomodulating adjuvant bacillus Calmette–Guérin vaccine. For patients with stage II disease, ASI not only significantly prolonged the recurrence-free interval but also improved the 5-year overall survival and recurrence-free survival. Nonetheless, a statistically significant prognostic effect was not achieved in patients with stage III disease [64]. Pembrolizumab, an antibody targeting programmed death receptor I, achieves its anti-tumor effects by bolstering autoimmunity. Moreover, 307 treatment-naïve patients with MSI-H or mismatch repair-deficient (dMMR) colon cancer were treated with pembrolizumab or chemotherapy. The former was better than the latter in terms of progression-free survival and overall remission rate. Furthermore, there were fewer treatment-related adverse events with pembrolizumab. This result signifies that pembrolizumab can be used as first-line treatment for MSI-H-dMMR metastatic colon cancer [7]. Edrecolomab is a mouse-derived monoclonal IgG2a antibody that specifically binds to antigens associated with human tumors. In studies involving patients with resected stage III colorectal cancer, edrecolomab treatment resulted in a 32% increase in overall survival and a 23% reduction in the rate of tumor recurrence compared with the untreated group [65]. The administration of edrecolomab is a novel immunotherapeutic strategy that holds promise as the preferred monotherapy for stage II colon cancer and the combination chemotherapeutic regimen for stage III colon cancer.

### 3.4. Radiotherapy

**Figure 1:**
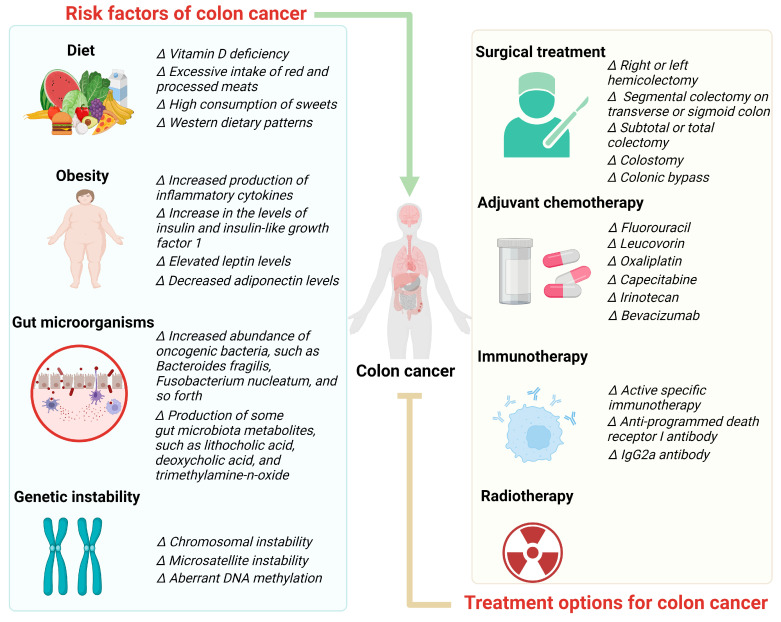
Risk factors and treatments of colon cancer.

Although adjuvant radiotherapy is not routinely recommended for patients with colon cancer, it is indicated in those with a high risk of local recurrence, such as advanced local disease and/or positive margins. Patients with localized lesions in advanced stages and those with positive resection margins exhibited improved overall survival when radiotherapy was administered [66]. Randomly assigned patients with colon cancer were treated with fluorouracil and levamisole (levamisole, the levorotatory isomer of tetramisole, restores the function of suppressed macrophages and T cells to their normal state), followed by radiotherapy in one group and no radiotherapy in the other group. The results revealed that the 5-year overall survival rates for patients treated with chemotherapy alone and those treated with a combination of chemotherapy and radiotherapy were 62% and 58%, respectively. The 5-year disease-free survival rate was 51% in both groups, which was not significantly different. However, radiation therapy resulted in increased toxicity [67]. Risk factors and the treatment of colon cancer are shown in Figure 1.

## 4. Curative Effects and Mechanisms of Action of Probiotics on Colon Cancer

### 4.1. Inhibition of Proliferation and Induction of Apoptosis in Cancer Cells

Cytotoxicity assay revealed that colon cancer cell growth was suppressed following the application of probiotics. *Lacticaseibacillus casei* ATCC393 inhibited the growth of CT26 and HT-29 cells by 52% and 78%, respectively, after 24 h of treatment at a concentration of 10^9^ CFU/mL [68]. The sulforhodamine B colorimetric assay indicated that treatment with *Pediococcus pentosaceus* SP2 at 10^8^ CFU/mL for 48 h reduced the viability of HT-29 cells by 80% [8]. The methylthiazolyldiphenyl-tetrazolium (MTT) assay (a method for measuring cell viability) showed that when the probiotic *Pediococcus pentosaceus* GS4 acted on HCT-116 cells, cell viability was only 32.66% [9]. Following the treatment of colon cancer cells with *Lacticaseibacillus casei* JY300-8, the impact of Lacticaseibacillus casei JY300-8 on cell proliferation activity was assessed using the MTT assay. This suppressed the growth of Caco-2 cells by up to 65.27% and that of HT-29 and HCT-116 cells by >80% [69]. The treatment of HT-29 cells with the probiotic mixture of *Lactobacillus sporogenes* and *Clostridium butyricum* and the drug 5-FU resulted in 57% and 68% cell survival, respectively, after 72 h, whereas the combination of probiotics and drugs resulted in a cell survival rate of only 38% [70]. *Lactobacillus acidophilus* KLDS1.0901 was used to treat HT-29 cells at different multiplicities of infection (MOI, rate of *Lactobacillus* to cells), i.e., 1, 10, 50, and 100, and the treatment durations were 12, 24, and 48 h. The maximum inhibition of HT-29 cells at MOI = 100 was 40.51% at 48 h [71]. Moreover, these live probiotics exhibited differing inhibitory effects on colon cancer cells at varying concentrations and time periods. Also, the inhibitory effect of inactivated probiotics on colon cancer has been experimentally examined. The growth of HT-29 cells was considerably suppressed after treatment with heat-inactivated *Levilactobacillus brevis* and *Lacticaseibacillus paracasei* (heated at 80 °C for 30 min). The inhibitory effect of *Levilactobacillus brevis* was stronger than that of *Lacticaseibacillus paracasei*, and there was no significant toxicity to normal HEK-293 cells [72]. After the heat inactivation of *Saccharomyces cerevisiae*, the survival rate of SW480 cells decreased to 28.49%, and the inhibitory effect was higher than that of the anticancer drug 5-FU [73]. Hence, microbial viability may not have an effect on the probiotic’s ability to suppress the growth of cancer cells.

Flow cytometry analysis revealed that the colon cancer cell cycle was altered after probiotic treatment and that the proportion of cells in the G_1_ phase increased. According to the findings, 51.69% of the cells were blocked in G_1_ phase after *Lacticaseibacillus paracasei* X12 treatment. The treatment further upregulated p27, a cell cycle protein-dependent kinase inhibitor involved in the mTOR/4E-BP1 pathway, which blocked the cell cycle [74]. In addition, the expression levels of certain cell cycle-related genes were altered during the process. The expression levels of the cell cycle proteins cyclin A, cyclin B_1_, cyclin B_2_, cyclin D_1_, and cyclin E were significantly downregulated [68,75]. In other studies, chromatin condensation and nuclear fragmentation were observed upon treating colon cancer cells with probiotics (*Lactobacillus sporogenes*, *Clostridium butyricum*, and *Saccharomyces cerevisiae*), which implies the occurrence of apoptosis [70,73]. Apoptosis was further confirmed by staining the cells with Annexin V-FITC/PI. A significant increase in the proportion of early and late apoptotic cells was noted, with >90% of cells undergoing apoptosis [68]. The treatment of HT-29 cells with *Lactobacillus acidophilus* KLDS1.0901 dramatically increased the number of apoptotic cells compared with untreated cells, which reduced the mitochondrial membrane potential and increased the ROS levels [71]. The apoptosis-promoting genes Bax, Bid, Bad, and Bak and the apoptosis inhibitor genes Bcl-2 and Bcl-XL were altered during apoptosis [76,77]. *Levilactobacillus brevis* and *Lacticaseibacillus paracasei* enhanced the mRNA expression levels of Bax, caspase-3, and caspase-9 and inhibited the expression of Bcl-2 in HT-29 cells by initiating the mitochondrial pathway, thereby promoting apoptosis [72]. The administration of *Lacticaseibacillus rhamnosus* GG and *Lactobacillus acidophilus* decreased the tumor incidence, tumor load, and tumor recurrence in a 1,2-dimethylhydrazine (DMH)-induced colon tumor model. These two strains induced apoptosis by downregulating the expression of the proto-oncogene K-ras and upregulating the expression of p53 [10]. After the induction of colon cancer in mice with the drug azoxymethane (AOM), *Pediococcus pentosaceus* GS4 reduced disease severity and tumor characteristics and also lowered the ROS levels to alleviate oxidative stress. Furthermore, GS4 inhibited the expression of nuclear factor κB (NF-κB) and p-Akt, which promoted apoptosis via the Akt/NF-κB signaling pathway [9].

Taken together, probiotics suppress the development of colon cancer by downregulating the genes encoding cell cycle proteins to block the cell cycle and upregulating the expressions of genes and proteins involved in the apoptotic process.

### 4.2. Immunomodulation

Probiotics prevent disease via immunomodulation, which is a common mechanism. Probiotics can relieve colon cancer by modulating inflammatory factors and chemokines, increasing the number of T-cells, producing immunoglobulins, and activating phagocytosis.

Colon cancer requires the involvement of certain inflammatory cytokines, such as TNF-α, IL-1β, IL-6, and IL-17. On the contrary, some anti-inflammatory factors, such as interferon (IFN)-γ and IL-10, can mitigate colon cancer. Oral treatment with *Lacticaseibacillus casei* significantly reduced the tumor volume in mice. Cytokine production, as determined using an enzyme-linked immunosorbent assay, revealed considerable elevation in the levels of IFN-γ, IL-12, and IL-10 [78]. The probiotics *Lactobacillus coryniformis* MXJ32 and *Clostridium butyricum* reduced the occurrence of colonic lesions and attenuated the severity of the disease in colon cancer mouse models by enhancing the expression of anti-inflammatory cytokines and reducing the expression of proinflammatory cytokines [77,79]. DMH induced colon cancer in rats, resulting in a significant increase in the expression of cyclooxygenase-2 (COX-2). COX-2 catalyzes the production of prostaglandins from arachidonic acid, thereby stimulating cell proliferation and proinflammatory processes. However, the administration of probiotics *Lactiplantibacillus plantarum* and *Lacticaseibacillus rhamnosus* GG resulted in reduced COX-2 expression [80].

Colon cancer was induced in mice via the subcutaneous injection of CT26 cells, and the animals were treated with the oral administration of *Lacticaseibacillus casei*. The findings indicated a distinct increase in tumor-infiltrating lymphocytes and an increase in the ligands for the CC chemokine receptor 5 (CCR5) and CXC chemokine receptor 3 (CXCR3). This observation could be ascribed to the generation of Th1 immunostimulatory cytokines and chemokines in tumor tissues as well as the transference of T cells in tumors [78]. In addition, treatment with *Lactiplantibacillus pentosus* B281 and *Lactiplantibacillus plantarum* B282 caused a significant infiltration of leukocytes in mice with colon cancer, and most of the infiltrating leukocytes were neutrophils. Both strains increased the expressions of IL-1α, IL-1β, IL-6, chemokine (C-X-C motif) ligand (CXCL)-1, chemokine (C-C motif) ligand (CCL)-3, CCL-4, and CXCL-2 and decreased the expression of the macrophage colony-stimulating factor [81]. In addition, probiotics *Lactobacillus acidophilus* significantly enhanced the number of CD8^+^ and CD4^+^ cells in mice with AOM-induced colon cancer [82]. Along with an increase in the number of CD8^+^ cells, apoptosis in the tumor tissue also demonstrated an increase in mice with tumors treated with probiotics [83]. Furthermore, *Lactobacillus acidophilus* substantially reduced the expression levels of the tumor markers carbohydrate antigen 19-9 and carcinoembryonic antigen (CEA) [82]. This finding alludes that A probiotic can inhibit colon cancer via T cell-mediated immune response. Also, probiotics stimulate the body to produce immunoglobulin A (IgA), which reduces the exposure of colon cells to certain carcinogens [84]. In a clinical trial, administering oral bifid triple viable probiotics to patients undergoing radical colorectal resection was shown to bolster host immunity, elevate serum IgG and IgA levels, and diminish postoperative infective complications [85]. Patients undergoing colon resection who received preoperative oral *Saccharomyces boulardii* exhibited lowered mucosal levels of IL-1β, IL-10, and IL-23A [86]. Additionally, probiotics modulated the immune response via the induction of phagocytosis, which inhibited cancer cells at an early stage of development [87].

### 4.3. Regulation of Intestinal Microorganisms and Their Metabolism

Dysregulation of gut microbial ecology can lead to alterations associated with colon cancer in the host physiology. Studies have established that probiotics can ameliorate colon cancer by modulating the gut microbiota. Among other modes of action, probiotics mitigate colon cancer by augmenting the number of salutary microorganisms, inhibiting pernicious microorganisms, and altering the metabolic activity of intestinal metabolites and intestinal flora.

In mice models of colon cancer induced by DMH or AOM/dextran sulfate sodium (DSS), probiotic treatment resulted in changes to microbial diversity and abundance. It also induced alterations in microbiota composition, facilitating the restoration of a healthy gut microbiota state [69,76,88]. Supplementation with *Loigolactobacillus coryniformis* MXJ32 augmented *Lactobacillus*, *Bifidobacterium*, *Akkermansia*, and *Faecalibaculum*. These genera exhibit a positive correlation with colon length, goblet cells, and short-chain fatty acids, potentially contributing to the alleviation of colon cancer. Conversely, the quantities of *Desulfovibrio* and *Helicobacter* decreased, which can exacerbate intestinal inflammation [79]. Likewise, supplementation with *Clostridium butyricum* decreased the *Firmicutes*/*Bacteroidetes* ratio at the phylum level [77]. *Firmicutes* and *Bacteroidetes* represent the two predominant phyla within the gut microbiota and serve as indicators of microbial equilibrium. The application of *Ligilactobacillus salivarius* Ren decreased the abundance of *Ruminococcus* species, *Clostridiales*, and *Bacteroides dorei* [88]. *Bacteroides* and *Clostridiales* have been linked to the onset of colon cancer. Similar results were also observed in clinical trials. Patients who underwent the resection of anterior sigmoid colon cancer were treated with a probiotic powder containing *Bifidobacterium animalis* subsp. *lactis* HY8002 (1 × 10^8^ CFU), Lacticaseibacillus casei HY2782 (5 × 10^7^ CFU), and *Lactiplantibacillus plantarum* HY7712 (5 × 10^7^ CFU) for 4 weeks. The findings showed that harmful bacteria related to colon cancer (*Alloprevotella* and *Porphyromonas*) decreased after the probiotic treatment [89]. These two detrimental bacteria are linked to an elevation in pro-inflammatory factors.

After the induction of colon cancer in mice with AOM/DSS, the gavage of *Bifidobacterium bifidum* CGMCC 15068 led to a reduction in tumor incidence by altering not only the microbiota composition but also intestinal metabolites [90]. In this study, some metabolites involved in the tricarboxylic acid (TCA) cycle, glycolysis, butyric acid metabolism, fatty acid biosynthesis, and other processes were considerably altered [90]. Of the various metabolic pathways, the TCA cycle and glycolysis play key roles in cancer. Butyrate is a vital metabolite for maintaining intestinal homeostasis and exerts anticancer and anti-inflammatory activities. The abundance of butyrate-producing bacteria-*Akkermansia* increased after the administration of CGMCC 15068 [90]. These findings imply that the gut microbiota may play a role in modulating changes in the metabolome, potentially influencing both colon health and the progression of colon cancer. Similar outcomes have been observed in clinical trials. For instance, in a 12-week trial administering the probiotic *Lactobacillus gasseri* OLL2716: LG21, researchers noted a reduction in fecal pH and fecal spoilage products, along with an increase in the short-chain fatty acid isobutyrate [91]. Additionally, oral probiotics have been shown to reduce serum endotoxin and D-lactic acid levels [85]. Gut microbiota produce enzymes such as β-glucosidase and β-glucuronidase, which catalyze the conversion reactions of polycyclic aromatic hydrocarbons, heterocyclic aromatic amines, primary bile acids, etc., to produce active carcinogens and generate several toxic metabolites that can lead to cancer [92]. Urease catalyzes the hydrolysis of urea to produce ammonia, whose derivatives are extremely poisonous and are capable of causing mutagenic DNA damage [93]. When healthy subjects were administered capsules containing live microorganisms (*Lacticaseibacillus rhamnosus* LC705 and *Propionibacterium freudenreichii* ssp. *shermanii* JS, 2 × 10^10^ CFU of each strain daily), the activities of fecal β-glucosidase and urease were reduced by 10% and 13%, respectively. Furthermore, the decrease in β-glucosidase activity was linked to an increase in *Propionibacterium* [94]. The reduced activity of β-glucuronidase was also observed after rats with colon cancer were gavaged with probiotic fermented milk [95]. This finding signifies that probiotics alter the growth and metabolism of microorganisms by inhibiting the activities of metabolic enzymes, thereby reducing the risk of colon cancer.

### 4.4. Strengthening of the Intestinal Barrier

The intestinal barrier maintains intestinal homeostasis and prevents the displacement of the intestinal microbiota and leakage of the intestinal material. However, the intestinal barrier function is often impaired and the intestinal environment deteriorates in patients with colon cancer. Transmembrane proteins are present between intestinal epithelial cells and bind to the cytoskeleton to form tight junctions. Increased intestinal permeability during inflammation and cancer alters the expressions of these proteins [96]. The gavage of *Lactiplantibacillus plantarum* KX041 isolated from kimchi in AOM/DSS-induced colon cancer mice reduced the intestinal leakage and increased expression levels of occludin, claudin-1, and ZO-1 [97]. The possible reason is that probiotics reduce the absorption of carcinogenic and inflammatory substances and protect colon cells. Furthermore, treatment with the probiotic capsule containing *Lactiplantibacillus plantarum* CGMCC 1258, *Lactobacillus acidophilus* LA-11, and *Bifidobacterium longum* BL-88 augmented transepithelial resistance and mucosal tight junction protein expression levels [98]. The administration of *Loigolactobacillus coryniformis* MXJ32 significantly suppressed the total tumor number and mean tumor diameter in mice after the AOM/DSS induction of colon cancer. Moreover, MXJ32 restored the reduction in goblet cells [79]. *Lacticaseibacillus rhamnosus* LS8 significantly attenuated inflammatory infiltration and crypt damage in colon cancer mice, and the mortality rate was reduced from 35% to 25% and the colon length was increased by 9.21%. In another study, LS8 attenuated AOM/DSS-induced goblet cell loss and promoted the expression of tight junction proteins [99]. Mucins produced by goblet cells are key components of the intestinal barrier and reduce the possibility of carcinogens and pathogens coming into contact with colon cells. Therefore, decreased mucin production increases colon cancer risk [100]. Hence, probiotics may alleviate symptoms of colon cancer by enhancing the expression of tight junction proteins and restoring the loss of goblet cells.

### 4.5. Enhancement of Antioxidant Activity

**Figure 2:**
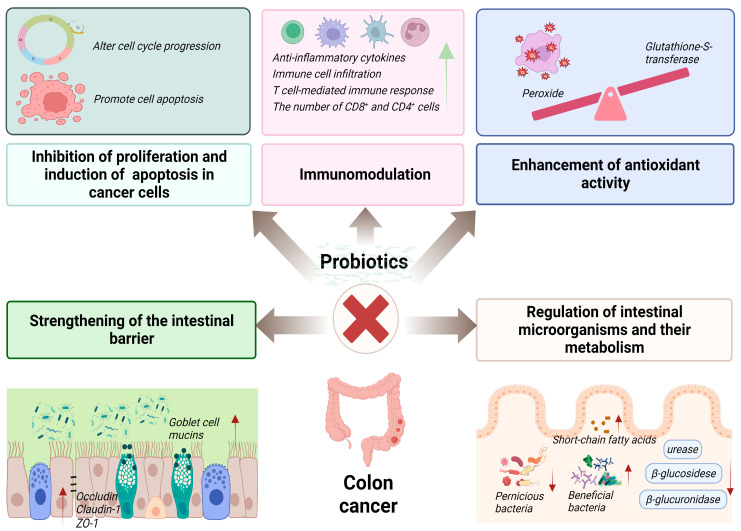
The action mechanisms of probiotics in the prevention of colon cancer.

ROS accumulation and oxidoreductase enzyme imbalance can lead to tumor development. Some ROS (e.g., hydrogen peroxide) influence colon cancer cell growth and invasion. However, tumor development can be altered by regulating the activity of antioxidant enzymes, thereby reducing the spread and growth of colon cancer. The probiotic fermented milk containing *Lactobacillus acidophilus* CL1285, *Lacticaseibacillus casei* LBC80R, and *Lacticaseibacillus rhamnosus* CLR2 exerted a therapeutic effect on colon cancer by reducing the number of abnormal crypt foci in colon cancer rats. The mechanism of action was related to an increase in the activity of glutathione-S-transferase with detoxifying effects [95]. The probiotic *Lactiplantibacillus plantarum* AS1, derived from the traditional fermented food Kallappam, was examined for its ability to inhibit peroxidation and scavenge free radicals in vitro. Results indicated that *Lactiplantibacillus plantarum* AS1 exhibited a 50.96% inhibition rate of linoleic acid peroxide and a 29.15% elimination rate of DPPH free radicals. Moreover, in a DMH-induced rat model of colon cancer, rats administered with *Lactiplantibacillus plantarum* AS1 demonstrated reduced tumor incidence and a decrease in the mean number of tumors. Comparatively, the control group exhibited significantly increased levels of lipid peroxidation in both colon and plasma following the DMH treatment. However, after probiotic treatment, lipid peroxidation levels returned to normal, suggesting that *Lactiplantibacillus plantarum* AS1 regulates colon cancer development through its antioxidant properties [101]. The mechanisms of probiotics in the treatment of colon cancer are summarized in Figure 2.

## 5. Curative Effects of Probiotic Components and Metabolites on Colon Cancer

### 5.1. Probiotic Metabolites

The cell-free supernatant of *Bifidobacterium* caused cytopathic effects, such as fragmentation, cell destruction, and decreased cell density in SW742 cells, significantly inhibiting cell proliferation [102]. *Kluyveromyces marxianus* AS41 was isolated and characterized from cheese. Treatment with its cell-free supernatant resulted in 26.7% and 33.1% survival of HT-29 and Caco-2 cells, respectively, but no significant toxicity to KDR/293 cells. Additionally, the proportion of inchoate and advanced apoptotic cells increased [103]. The probiotic *Pichia kudriavzevii* AS-12 supernatant acted on HT-29 and Caco-2 cells and induced apoptosis. The reduction in cell volume, condensation, or fragmentation of nuclei, and the formation of membrane vesicles and apoptotic vesicles were observed. Apoptosis was induced by upregulating the expressions of Bad, caspase-9, caspase-3, caspase-8, and ligand Fas R and downregulating the expression of Bcl-2 [104]. In addition, probiotic supernatants inhibited the activity of matrix metalloproteinase 9 (MMP-9) linked to cancer risk. *Lacticaseibacillus rhamnosus*, *Lactobacillus crispatus*, *Lactobacillus delbrueckii*, and *Limosilactobacillus reuteri* supernatants inhibited cancer cell proliferation, promoted apoptosis, and downregulated the expression of MMP-9 to reduce cancer cell invasion [12,105,106]. Treating Caco-2 cells with different concentrations of *Saccharomyces cerevisiae* var. *boulardii* metabolites for 24 and 48 h showed that 2000 μg/mL exerted the best inhibitory effect after 24 h of treatment. Metabolites of *Saccharomyces boulardii* induced apoptosis and further inhibited the mRNA expressions of genes encoding IL-8 and NF-κB [107]. Thus, probiotic metabolites can arrest the progression of colon cancer. However, the exact compounds in these probiotic supernatants that exert apoptotic effects are yet to be elucidated, and further studies focusing on the supernatant components and evaluating their effects on cancer cells are required.

Nisin is a peptide substance produced by *Lactococcus lactis* and is an important metabolite. At 40–50 IU/mL, nisin significantly suppressed the proliferation of LS180 cells, and at 250–350 IU/mL, it inhibited the growth of SW48, HT-29, and Caco-2 cells. The expressions of the metastatic genes CEA, CEAM6, and MMP2F were significantly downregulated after the treatment with nisin [108]. A bacteriocin-producing probiotic strain, *Pediococcus acidilactici* K2a2-3, was isolated and identified from the intestine. HT-29 cells were treated with pediocin produced by the K2a2-3 strain. At concentrations of 800 AU/mL and 1600 AU/mL, the inhibitory rates against cancer cells were 55% and 53.7%, respectively [109]. Plantaricin P1050 was identified from the *Lactiplantibacillus plantarum* PBS067 strain, and used to treat healthy colon CCD 841 cells and E705 colon cancer cells at a concentration of 1 µg/mL for 48 h. The results revealed a 20% increase in the viability of healthy colon cells and a 30% decrease in colon cancer cell viability [110]. However, the molecular mechanism underlying the inhibition of colon cancer cell proliferation by plantaricin P1050 requires further investigation. Additionally, a novel peptide (Entap) with anti-proliferative effects on HT-29 cells was isolated from the bacteria of the *Enterococcus* genus. This peptide may inhibit colon cancer by disrupting the cell cycle and inducing autophagy [111]. The cell-free supernatant of the *Lacticaseibacillus casei* ATCC334 culture simultaneously suppressed the proliferation of Caco-2/bbe, SKCO-1, and SW620 cells. One of the tumor suppressor molecules was analyzed and identified as ferrochrome via mass spectrometry. Ferrochrome in the supernatant specifically inhibited the proliferation of cancer cells without affecting intestinal epithelial cells. The cancer inhibitory effects of 5-FU and cisplatin are well known, and the effects of ferrochrome were either superior to or comparable to these drugs. Ferrochrome treatment induced massive apoptosis, which was reduced when the c-Jun N-terminal kinase (JNK) signaling pathway was inhibited. This finding suggests that the probiotic metabolite ferrochrome acts by activating the JNK signaling pathway [112]. Butyrate is a key metabolite in microorganisms. Butyrate administration in the mice with AOM/DSS caused a decrease in the number of tumors, the disappearance of intestinal wall thickening, and a significant reduction in colon damage, bleeding, and inflammation. Furthermore, butyrate promoted the colonization of *Actinobacteriota* (antibiotic-producing bacteria), *Bifidobacteriales* (key symbiotic bacteria), and *Muribaculacea* (intestinal mucus-producing symbiotic bacteria), which are beneficial microorganisms [113]. The supernatant containing butyrate from *Lactiplantibacillus plantarum* S2T10D significantly inhibited the growth of HT-29 cells, arrested the cell cycle at the G_2_/M phase, and reduced the expression levels of cyclin D_1_ and cyclin B_1_. Conversely, the non-butyrate-producing strain O2T60C exhibited a limited ability to inhibit the growth of HT-29 cells. Viable strains S2T10D and O2T60C inhibited HT-29 cell growth. But the inhibitory effect of S2T10D was greater than that of the other strain, probably due to its higher butyric acid-producing capacity [114].

### 5.2. Probiotic EPSs

EPSs have received much attention because of their antioxidant, immunomodulatory, and anticancer activities. The EPSs of probiotics have few side effects and therefore could be a potential new source of anticancer drugs. EPSs of probiotics exhibited anticancer potential in vitro, significantly restraining the viability of cancer cells and inducing apoptosis. The EPS-producing strain *Limosilactobacillus fermentum* YL-11 was isolated from dairy products, and the inhibition rate of EPSs at 600 μg/mL for 48 h on HT-29 cells was 46.5% [115]. EPSs from *Lactobacillus delbrueckii* subsp. *lactis* NCDC252 inhibited HCT-116 cells up to 67.1% after 48 h of action at 10 μg/mL [116]. The antiproliferative activity of EPSs from *Lacticaseibacillus paracasei* increased from 36% to 80% after treating HT-29 cells at 40 mg/mL for 24 and 72 h, and the maximum apoptosis-inducing effect was observed at 48 h [117]. This observation implies that the effects of probiotic EPSs on antiproliferative activity and apoptosis-inducing activity are dose-, time- and strain-dependent. EPSs from *Lacticaseibacillus paracasei* caused remarkable early/late apoptotic cell death after incubation at 15 μg/mL for 24 h. The findings showed that 41.16%, 41.86%, and 44.74% of SW-480, HT-29, and HCT-116 cells, respectively, were in the early/late apoptotic or necrotic phase. EPSs from *Lacticaseibacillus paracasei* blocked the mRNA expression of Akt-1, mTOR, and Janus kinase (Jak)-1, reduced the level of Bcl-2, and enhanced the levels of Bax, caspase-3, and caspase-8 [118]. EPSs of *Streptococcus thermophilus* and *Lactobacillus delbrueckii* subsp. *bulgaricus* from yogurt displayed good anticancer activity. Moreover, the activities of EPSs were significantly enhanced when the concentration was increased within a certain range. The inhibitory effects of EPSs from *Streptococcus thermophilus* and *Lactobacillus delbrueckii* subsp. *bulgaricus* on HT-29 cells after 24 h of treatment at 5 mg/mL were 32.28% and 53.06%, respectively, and were 89.52% and 60.70% on HCT-116 cells, respectively. In addition, EPSs exerted good antioxidant activity, as evidenced by their ability to scavenge the free radicals DPPH and ABTS [13]. LHEPS-1, the EPS fraction purified from *Lactobacillus helveticus* MB2-1, acted at 600 μg/mL for 72 h to inhibit Caco-2 cells by 56.34%. Further analysis of EPSs from MB2-1 revealed that their structural characteristics, such as monosaccharide composition, molecular weight, conformation, and branching degree, affected their anti-colon cancer activity [119]. EPSs have also been observed to alleviate colon cancer in in vivo experiments. EPSs from *Limosilactobacillus fermentum* YL-11 inhibited tumor growth and reduced the tumor weight in colon cancer mice. The anti-colon cancer mechanism may be related to the activation of endogenous mitochondrial caspases and the inhibition of the PI3K/AKT signaling pathway as well as the regulation of cell cycle-related proteins [120]. The supplementation of EPSs from *Lactiplantibacillus plantarum*-12 alleviated colon cancer symptoms by restoring colon length and significantly reducing tumor numbers in mice with AOM/DSS-induced colon cancer [121].

### 5.3. Probiotic Slp

The Slp is situated in a paracrystalline layer outside the bacterial cell wall. Slp plays a crucial part in preserving the cellular morphology and structure, promoting intercellular adhesion, recognizing external receptors, preventing the attachment of pathogenic bacteria, and regulating the immune response. Furthermore, probiotic Slp has been reported to exert anticancer effects. The Slp from *Lactobacillus acidophilus* CICC 6074 inhibited the proliferation of HT-29 cells, and the survival rate of the cells was 54.68% when the concentration of Slp reached 100 mg/L. Upon Slp action, the levels of p53, p21, and p16 were upregulated, whereas those of cyclin-dependent kinase and cyclin B were downregulated, which arrested the cell cycle in the G_0_/G_1_ phase. After the Slp treatment, the cells showed obvious changes characteristic of apoptosis (e.g., chromatin condensation, nuclear fragmentation, and vacuole formation). In addition, Slp remarkably elevated the mRNA levels of Fas, FADD, caspase-3, caspase-8, Bax, and caspase-9 and decreased the level of Bcl-2. A reduction in cellular mitochondrial membrane potential was also noted. The results suggested that Slp may induce apoptosis by activating the death receptor pathway and interfering with the mitochondrial pathway [14]. The Slp of *Lactobacillus acidophilus* NCFM significantly suppressed the growth of HCT116 cells and further caused cellular autophagy. The expressions of autophagy proteins Atg7, LC3-II, and Beclin-1 were increased in HCT-116 cells, and autophagic vacuoles were formed. The results demonstrated that Slp promoted ROS accumulation, suppressed mTOR expression, and activated JNK activity, thereby inducing autophagy [122]. In view of its anticancer activity, the Slp of *Lactobacillus acidophilus* may serve as a potential anticancer drug. In the future, in vivo confirmation of its inhibitory effect on colon cancer is warranted.

**Figure 3:**
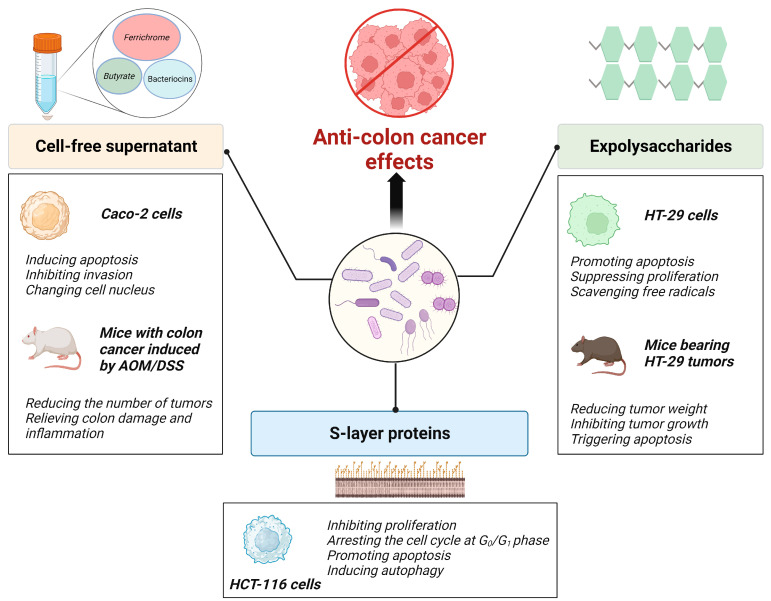
Anti-colon cancer effects of probiotic components and metabolites.

Both live and inactivated forms of probiotics exhibit anti-colon cancer activity. Furthermore, other components with probiotic properties, such as secreted metabolites and EPSs, as well as Slp exert inhibitory effects on colon cancer. The anti-colon cancer effects of probiotic components and metabolites are summarized in Figure 3.

This review provides a summary of research on the inhibition of colon cancer by probiotics, probiotic components, and metabolites. Cellular experiments are detailed in Table 1, animal experiments are detailed in Table 2, and clinical studies are detailed in Table 3.

## 6. Conclusions

The factors influencing the occurrence of colon cancer are complex and multifaceted. Key contributors include diet, obesity, the composition of the intestinal microbiome, and genetic instability, all of which promote colon cancer. Current mainstream treatment options for colon cancer patients encompass surgery, chemotherapy, immunotherapy, and radiation therapy. However, it is important to acknowledge that these treatments come with limitations and side effects. Substantial research evidence highlights the pivotal role of probiotics in preventing and alleviating colon cancer. This review illuminates the diverse mechanisms through which probiotics contribute to mitigating colon cancer, including inhibiting cell proliferation and promoting apoptosis, enhancing the immune response, regulating the intestinal microbiota and their metabolic processes, strengthening the intestinal barrier, and boosting antioxidant effects. Notably, probiotics themselves exhibit anticancer activity, and the metabolites they produce, such as bacteriocins and short-chain fatty acids, possess the capacity to inhibit colon cancer. Additionally, certain bacterial components, such as EPSs and Slp, also play an inhibitory role in colon cancer.

## 7. Future Prospects

Probiotics exert their anticancer activity via physiological mechanisms that are usually strain specific. Therefore, further investigations should target these strains with anti-cancer activity to determine the optimal dose, duration, and frequency of treatment. Research into the development of probiotic products (e.g., probiotic capsules and yogurt) for individuals at a high risk of developing colon cancer is one of the avenues for future work in the field. In the past, there have been few reports on whether oral probiotics have side effects, which is a promising direction for further research. In addition, more randomized, double-blind, and placebo-controlled clinical trials should be conducted to validate the efficacy of probiotics in treating colon cancer.

## Figures and Tables

**Table 1:** In vitro studies of probiotics, their components, and metabolites inhibiting colon cancer.

Cells	Probiotics, Their Components, and Metabolites	Food Sources of Probiotics	Treatments	Results	Ref.
Caco-2 cells	Conditioned media for *Lactiplantibacillus pentosus* B281 and *Lactiplantibacillus plantarum* B282	Naturally fermented olives	Cells were treated with different concentrations of probiotic-conditioned media for 24, 48, and 72 h at 37 °C.	Inhibition of cell proliferation; decreased colony formation; cell cycle arrest in the G_1_ phase; decreased expression of cyclin A, cyclin B_1_, cyclin B_2_, and cyclin E, and increased expression of cyclin D.	[75]
CT26 and HT-29 cells	*Lacticaseibacillus casei* ATCC393	NA	Cells were treated at a concentration of 10^8^ CFU/mL for 24, 48, and 72 h at 37 °C.	Inhibition of cell proliferation; promotion of apoptosis; increased expression of the apoptosis-inducing ligand TRAIL and decreased expression of cyclin D_1._	[68]
HT-29 cells	Heat-inactivated *Levilactobacillus brevis* and *Lacticaseibacillus paracasei*	Iranian food “Terxine”	Cells were treated with different concentrations of probiotics for 24, 48, and 72 h at 37 °C.	Inhibition of cell proliferation; activation of mitochondrial pathway to promote apoptosis.	[72]
SW480 cells	Heat-inactivated *Saccharomyces cerevisiae*	NA	Cells were treated with different concentrations of probiotics for 24 and 48 h at 37 °C.	Inhibition of cell proliferation; activation of Akt/NF-κB signaling pathway to promote apoptosis.	[73]
HT-29 and HCT-116 cells	*Lactobacillus sporogenes* and *Clostridium butyricum* TO-A	NA	Cells were treated at 10^7^ CFU/mL for 24, 48, and 72 h at 37 °C.	Inhibition of cell proliferation; promotion of apoptosis; increased expression of Bax, Bid, Bad, and Bak and decreased expression of Bcl-2 and Bcl-XL.	[70]
Caco-2 cells	*Saccharomyces cerevisiae* var. *boulardii* CNCM I-745 metabolites	NA	Cells were treated with different concentrations of metabolites for 24 and 48 h at 37 °C.	Inhibition of cell proliferation; promotion of apoptosis; inhibition of IL-8 and NF-κB gene expression.	[107]
HT-29 and Caco-2 cells	*Lactobacillus acidophilus* KLDS1.0901	NA	Cells were treated with different concentrations of probiotics for 12, 24, and 48 h at 37 °C.	Inhibition of cell proliferation; promotion of apoptosis; decreased cellular mitochondrial membrane potential; increased ROS levels.	[71]
CT26 cells	*Bifidobacterium longum*, *Bifidobacterium bifidum*, *Lactobacillus acidophilus*, and *Lactiplantibacillus plantarum*	NA	Cells were treated at 10^8^ CFU/mL for 24, 48, 72, and 96 h at 37 °C.	Inhibition of cell proliferation, migration, and invasion.	[83]
Caco-2, HT-29 and HCT-116 cells	*Lacticaseibacillus casei* JY300-8	Fresh corn stalks	Cells were treated with different concentrations of probiotics for 24, 48, 72, and 96 h at 37 °C.	Inhibition of cell proliferation; promotion of apoptosis.	[69]
SW48, HT-29, LS180 and Caco-2 cells	Nisin	NA	Cells were treated with different concentrations of nisin for 24 and 48 h at 37 °C.	Inhibition of cell proliferation; inhibition of cancer cell metastasis genes.	[108]
HT-29 and Caco-2 cells	*Pichia kudriavzevii* AS-12 cell-free supernatant	NA	Cells were treated with different concentrations of supernatants for 24 and 48 h at 37 °C.	Inhibition of cell proliferation; promotion of apoptosis; increased expression of Bad, caspase-3, caspase-8, caspase-9, and Fas-R and decreased expression of Bcl-2.	[104]
HT29-ShE cells	Heat inactivation of sonicated *Limosilactobacillus reuteri* and cell-free supernatants	NA	The 10% (*v*/*v*) heat-inactivated sonicated fraction and 5% supernatant treated cells for 24 h.	Inhibition of cell proliferation; promotion of apoptosis; inhibition of cancer cell metastasis; downregulation of MMP-9 and COX-2 expression.	[106]
Caco-2 and SW620 cells	Ferrochrome in the cell-free supernatant of *Lacticaseibacillus casei* ATCC334	NA	Treatment of cells with different concentrations of ferrochrome.	Inhibition of cell proliferation; induction of apoptosis through the activation of the JNK-DDTI3 pathway.	[112]
HT-29 and HCT-116 cells	EPSs of *Streptococcus pyogenes* and *Lactobacillus delbrueckii* subsp. *bulgaricus*	Labaneh (yogurt-like product)	Cells were treated with different concentrations of EPSs for 24, 48, and 72 h at 37 °C.	Inhibition of cell proliferation; facilitation of scavenging of the free-radicals DPPH and ABTS.	[13]
SW-480, HT-29 and HCT-116 cells	EPSs of *Lacticaseibacillus paracasei*	Dairy products	Cells were treated with different concentrations of EPSs for 24 and 48 h at 37 °C.	Inhibition of cell proliferation; promotion of apoptosis; increased expression of Bax, caspase-3, and caspase-8 and inhibition of AKT-1, JAK-1, and mTOR gene expression.	[118]
HT-29 cells	Slp of *Lactobacillus acidophilus* CICC 6074	NA	Cells were treated with different concentrations of Slp for 24 h at 37 °C.	Inhibition of cell proliferation; cell cycle block; promotion of apoptosis via death receptor pathway and mitochondrial pathway; inhibition of cell invasion.	[14]
HCT-116 cells	Slp of *Lactobacillus acidophilus* NCFM	NA	Cells were treated with different concentrations of Slp for 24 h at 37 °C	Inhibition of cell proliferation; cell cycle block; induction of autophagic death through inhibition of mTOR activity; activation of the JNK signaling pathway.	[122]

**Table 2:** In vivo animal model studies of probiotics, their components, and metabolites inhibiting colon cancer.

Animal Model	Probiotics, Their Components, and Metabolites	Food Sources of Probiotics	Treatments	Results	Ref.
A model of colon cancer induced by subcutaneous injection of CT26 cells; female BALB/c mice	*Lacticaseibacillus casei* ATCC393	NA	Probiotics (150 μL) at a dose of 10^9^ CFU/L were administered daily 10 days before subcutaneous injection of CT26 cells, for 13 days.	Tumor volume reduction; upregulation of TRAIL protein expression and downregulation of survivor protein expression in the tumor tissues.	[68]
DMH-induced colon cancer model in rats	*Lactiplantibacillus plantarum and Lacticaseibacillus rhamnosus* GG	Traditional fermented food of Himachal Pradesh	Probiotics were administered by gavage at a dose of 2 × 10^10^ CFU/day, once a week for 16 weeks, for 6 consecutive days.	Reduced tumor incidence, tumor diversity, and the mean tumor size; reduced total sialic acid and COX-2 expression.	[80]
DMH-induced colon cancer model; male Sprague–Dawley rats	*Lacticaseibacillus rhamnosus* GG and *Lactobacillus acidophilus*	NA	Probiotics were administered by gavage at a dose of 1 × 10^10^ CFU/day, one week prior to DMH administration for 18 weeks.	Decreased tumor load, tumor diversity; downregulation of Bcl-2 and K-ras gene expression, and upregulation of Bax and p53 gene expression.	[10]
Subcutaneous injection of CT26 cells induced colon cancer model; female BALB/c mice	*Lacticaseibacillus casei*	NA	Probiotics 150 μL at a dose of 10^9^ CFU/L were administered daily 10 days before subcutaneous injection of CT26 cells, for 13 days.	Decreased tumor volume; increased levels of IFN-γ, IL-12, and IL-10; increased tumor-infiltrating lymphocytes; increased expression of cleaved caspase-3 and cleaved PARP1.	[78]
AOM/DSS-induced colon cancer model; male C57BL/6 mice	*Loigolactobacillus coryniformis* MXJ32	Traditional fermented vegetable (Jiangshui Cai)	Probiotics were administered by gavage at a dose of 1 × 10^9^ CFU/day, for 15 weeks.	Suppression of total tumor number and average tumor diameter; restoration of intestinal barrier function; downregulation of inflammatory cytokine expression; increased amount of salutary bacteria and decreased amount of pernicious bacteria.	[79]
Colon cancer model induced by injection of CT26 cells; female BALB/c mice	*Bifidobacterium longum*, *Bifidobacterium bifidum*, *Lactobacillus acidophilus* and *Lactiplantibacillus plantarum*	NA	Probiotic mixture (1 × 10^9^ CFU/200 µL) administered daily via tube feeding, for 3 weeks.	Inhibition of tumor growth; increased apoptotic cells in tumor tissues; increased number of CD8^+^ cells.	[83]
AOM-induced colon cancer model; male BALB/c mice	*Lactobacillus acidophilus* and *Bifidobacterium bifidum*	Traditional yogurt	Probiotics (1 × 10^9^ CFU/g body weight per strain) were mixed in drinking water and gavaged daily for 10 days before AOM, for 5 months.	Decreased incidence of colonic lesions and smaller tumor size; decreased levels of tumor markers; increased levels of IFN-γ and IL-10; increased numbers of CD8^+^ and CD4^+^ cells.	[82]
AOM-induced colon cancer model; male C57BL/6 mice	*Bifidobacterium bifidum* CGMCC 15068	NA	Probiotics were administered by gavage at a dose of 3 × 10^8^ CFU/day during the recovery period.	Reduced tumor incidence; increased abundance of beneficial probiotics in the gut; altered gut metabolites.	[90]
Subcutaneous injection of CT26 cells induced colon cancer model; BABL/C male mice	*Lacticaseibacillus casei* JY300-8	Fresh corn stalks	Probiotics were administered by half-day gavage at a dose of 1 × 10^9^ CFU/mL until the end of the experiment.	Reduced tumor size and tumor incidence; decreased abundance of *Enterobacteriaceae* and *Clostridium perfringens*; increased abundance of *Lactobacillaceae* and *Bifidobacteriaceae*.	[69]
AOM/DMH-induced colon cancer model; male C57BL/6 mice	*Lactiplantibacillus plantarum* KX041	Traditional Chinese pickle juice	Oral administration of 200 μL probiotic suspension (1 × 10^9^ CFU/day), for 13 weeks.	Decrease in the total number and mean diameter of colon tumors; attenuation of inflammatory infiltration and crypt damage; increase in short-chain fatty acid content of feces; increased the number of salutary bacteria and decreased the number of pernicious bacteria.	[97]
DMH-induced colon cancer model; F344 male rats	*Lactobacillus acidophilus* CL1285, *Lacticaseibacillus casei* LBC80R and *Lacticaseibacillus rhamnosus* CLR2	NA	Gavage of 2, 1.5, 1, 0.5, and 0.25 mL of probiotic fermented milk daily, for 12 weeks.	Decreased counts of abnormal crypts in the colon; increased activity of glutathione-S-transferase and decreased activity of quinone reductase and β-glucuronide glycosidase.	[95]
Subcutaneous injection of CT26 cells induced colon cancer model; female BALB/c mice	EPSs of *Limosilactobacillus fermentum* YL-11	Fermented milk	Intravenous EPSs at 90 mg/kg body weight every alternate day, for 3 weeks.	Decreased tumor weight; nuclear consolidation; increased intracellular space; and fatty degeneration of tumor cells.	[120]
AOM/DSS-induced colon cancer model; male C57BL/6 mice	EPSs of *Lactiplantibacillus plantarum*	NA	EPSs at 200 mg/kg body weight/day via gavage, for 12 weeks.	Restoration of colon length and reduction in the tumor number; enhancement of intestinal barrier function; inhibition of the NF-κB pathway and activation of the caspase cascade reaction to promote apoptosis; alteration of the intestinal microbiota and metabolites.	[121]

**Table 3:** The clinical studies of probiotics inhibiting colon cancer.

Subjects	Probiotics	Food Sources of Probiotics	Treatments	Results	Ref.
22 patients undergoing radical colectomy	*Bifidobacterium longum*, *Lactobacillus acidophilus*, and *Enterococcus faecalis*	NA	Subjects received capsules containing live microorganisms 3 times/day (total daily dose of 6 × 10^7^ CFU).	Increased abundance and diversity of colonic mucosal microorganisms; decreased abundance of *Fusobacterium* and *Peptostreptococcus*.	[123]
60 patients who underwent resection of anterior sigmoid colon cancer	*Bifidobacterium animalis* subsp. *lactis HY8002*, *Lacticaseibacillus casei* HY2782, and *Lactiplantibacillus plantarum* HY7712	NA	Subjects received probiotic powder treatment twice daily, for 4 weeks.	Improved control of flatulence; increased abundance of beneficial bacteria; and decreased abundance of harmful bacteria in the gut.	[89]
10 colon cancer patients and 20 healthy subjects	*Lactobacillus gasseri* OLL2716: LG21	NA	Subjects administered probiotics daily, for 12 weeks.	Increased levels of *Lactobacillus* and decreased total *Clostridium perfringens* in the intestines; increased short-chain fatty acid isobutyrate in the feces; decreased pH; and decreased synthesis of spoilage products; increased NK cell activity.	[91]
38 men aged 24-55	*Lacticaseibacillus rhamnosus* LC705 and *Propionibacterium freudenreichii* ssp. *shermanii* JS	NA	Subjects consumed 2 capsules containing live microorganisms (2 × 10^10^ CFU per strain) per day, for 4 weeks.	Increased levels of *Lactobacillus* and *Propionibacterium* in the intestinal tract; decreased β-glucosidase and urease activity.	[94]
68 patients with treated colon cancer	*Lacticaseibacillus rhamnosus* R0011 and *Lactobacillus acidophilus* R0052	NA	Subjects were treated with probiotics (2 × 10^9^ CFU) daily, for 12 weeks.	Improved bowel symptoms and quality of life.	[11]
33 patients who underwent colectomy	*Saccharomyces boulardii*	NA	Subjects received preoperative probiotic oral therapy for at least 7 days.	Downregulation of mucosal IL-1β, IL-10, and IL-23A levels	[86]
60 patients who underwent radical colorectal resection	Bifid triple viable probiotics	NA	Subjects received oral probiotic treatment 3 days before surgery.	Reduced serum endotoxin, D-lactic acid, and IL-6 levels; elevated IgG and IgA levels; reduced incidence of postoperative infectious complications.	[85]

## Data Availability

No data were used for the research described in this article.

## Abbreviations

High microsatellite instability: MSI-H; extracellular polysaccharides: EPSs; S-layer protein: Slp; tumor necrosis factor-α: TNF-α; interleukin: IL; insulin-like growth factor 1: IGF-1; phosphatidylinositol 3-kinase: PI3K; mammalian target of rapamycin: mTOR; reactive oxygen species: ROS; enterotoxigenic *Bacteroides fragilis*: ETBF; adenomatous polyposis coli: APC; fluorouracil combined with leucovorin: FL; 5-fluorouracil: 5-FU; active specific immunotherapy: ASI; mismatch repair-deficient: dMMR; methylthiazolyldiphenyl-tetrazolium: MTT; multiplicities of infection: MOI; 1,2-dimethylhydrazine: DMH; azoxymethane: AOM; nuclear factor κB: NF-κB; interferon: IFN; cyclooxygenase-2: COX-2; CC chemokine receptor: CCR; CXC chemokine receptor: CXCR; chemokine (C-X-C motif) ligand: CXCL; chemokine (C-C motif) ligand: CCL; carcinoembryonic antigen: CEA; immunoglobulin A: IgA; dextran sulfate sodium: DSS; tricarboxylic acid: TCA; lipid peroxidation: LPO; matrix metalloproteinase: MMP; c-Jun N-terminal kinase: JNK; Janus kinase: Jak.
